# Mindfulness-based cognitive therapy *v.* treatment as usual in people with bipolar disorder: A multicentre, randomised controlled trial

**DOI:** 10.1017/S0033291723000090

**Published:** 2023-10

**Authors:** Imke Hanssen, Marloes Huijbers, Eline Regeer, Marc Lochmann van Bennekom, Anja Stevens, Petra van Dijk, Elvira Boere, Rob Havermans, Rogier Hoenders, Ralph Kupka, Anne E. Speckens

**Affiliations:** 1 Department of Psychiatry, Radboud University Medical Center, Center for Mindfulness, Reinier Postlaan 4, 6526 GC, Nijmegen, the Netherlands; 2 Donders Institute for Brain, Cognition, and Behaviour, Radboud University, Heyendaalseweg 135, 6525 AJ, Nijmegen, the Netherlands; 3 Altrecht Institute for Mental Health Care, Outpatient Clinic for Bipolar Disorders, Lange Nieuwstraat 119, 3512 PG, Utrecht, the Netherlands; 4 Pro Persona Mental Health Care, Outpatient Clinic for Bipolar Disorders, Nijmeegsebaan 61, 6525 DX, Nijmegen, the Netherlands; 5 Dimence Mental Health, Center for Bipolar Disorders, Pikeursbaan 3, 7411 GT, Deventer, the Netherlands; 6 PsyQ, Department of Mood Disorders, Lijnbaan 4, 2512 VA, The Hague, the Netherlands; 7 PsyQ, Department of Mood Disorders, Max Euwelaan 70, 3062 MA, Rotterdam, the Netherlands; 8 Department of Psychiatry, Leiden University Medical Center, Albinusdreef 2, 2333 ZA, Leiden, the Netherlands; 9 PsyQ Department of Bipolar Disorders, Mondriaan, Oranjeplein 10, 6224 KD, Maastricht, the Netherlands; 10 Lentis, Center for Integral Psychiatry, Hereweg 80, 9725 AG, Groningen, the Netherlands; 11 Department of Psychiatry, Amsterdam University Medical Center, Vrije Universiteit, Oldenaller 1, 1081 HJ, Amsterdam, the Netherlands

**Keywords:** Mindfulness-based cognitive therapy, bipolar disorder, randomised controlled trial, effectiveness

## Abstract

**Background:**

Mindfulness-based cognitive therapy (MBCT) seems a promising intervention for bipolar disorder (BD), but there is a lack of randomised controlled trials (RCT) investigating this. The purpose of this multicentre, evaluator blinded RCT was to investigate the added value of MBCT to treatment as usual (TAU) in BD up to 15 months follow-up (NCT03507647).

**Methods:**

A total of 144 participants with BD type I and II were randomised to MBCT + TAU (*n* = 72) and TAU (*n* = 72). Primary outcome was current depressive symptoms. Secondary outcomes were current (hypo)manic and anxiety symptoms, recurrence rates, rumination, dampening of positive affect, functional impairment, mindfulness skills, self-compassion, and positive mental health. Potential moderators of treatment outcome were examined.

**Results:**

MBCT + TAU was not more efficacious than TAU in reducing current depressive symptoms at post-treatment (95% CI [−7.0 to 1.8], *p* = 0.303, *d* = 0.24) or follow-up (95% CI [−2.2 to 6.3], *p* = 0.037, *d* = 0.13). At post-treatment, MBCT + TAU was more effective than TAU in improving mindfulness skills. At follow-up, TAU was more effective than MBCT + TAU in reducing trait anxiety and improving mindfulness skills and positive mental health. Exploratory analysis revealed that participants with higher depressive symptoms and functional impairment at baseline benefitted more from MBCT + TAU than TAU.

**Conclusions:**

In these participants with highly recurrent BD, MBCT may be a treatment option in addition to TAU for those who suffer from moderate to severe levels of depression and functional impairment.

**Trial registration:**

ClinicalTrials.gov, NCT03507647. Registered the 25 April 2018, https://www.clinicaltrials.gov/ct2/show/NCT01126827.

## Introduction

Bipolar disorder (BD) is one of the leading causes of disability (World Health Organization, 2008). Characterised by an early onset and a chronic course across the life span, BD is responsible for considerable economic, occupational, and social burden (Ferrari et al., 2016). A substantial number of people with BD experience recurrences or residual mood symptoms (Judd et al., 2003). Consequently, there is a need for additional interventions, including psychological interventions, not only targeting clinical remission, but also recurrence prevention, functional recovery, and quality of life. Mindfulness-based cognitive therapy (MBCT) is effective in reducing disorder-specific symptoms in a wide range of psychiatric disorders (Goldberg et al., 2018). In major depressive disorder, MBCT has shown to be efficacious in preventing depressive recurrences up to at least one year follow-up (Kuyken et al., 2016), and improving quality of life (Godfrin & van Heeringen, 2010). In BD this has not yet been sufficiently studied, although recent systematic reviews including 13 open-label and cohort studies conclude that MBCT holds promise, showing beneficial effects on current depressive and anxiety symptoms, and no destabilising effects on manic symptoms (Bojic & Becerra, 2017; Chu et al., 2018; Lovas & Schuman-Olivier, 2018; Xuan et al., 2020). To date, only two randomised controlled trials (RCT) investigating long-term effects of MBCT in BD have been conducted, in 95 and 84 people with BD respectively, showing no beneficial effects of MBCT on current symptoms or recurrence prevention when compared with treatment as usual (TAU) (Perich, Manicavasagar, Mitchell, Ball, & Hadzi-Pavlovic, 2013) or psychoeducation or TAU (de Dios et al., 2021). Results of these studies are limited by high drop-out rates (45%) at follow-up. Only one small cohort study (*N* = 12) investigated positive outcomes of MBCT in BD and showed improvements in well-being, positive affect and psychosocial functioning (Deckersbach et al., 2012). It is evident that there is a need for RCTs that investigate the additional effect of MBCT to TAU for BD. The current RCT aimed to investigate the efficacy of MBCT added to TAU *v.* TAU in BD, up to 15 months follow-up. Primary outcome was depressive symptoms at post-treatment. Secondary outcomes were long-term depressive symptoms, and both post-treatment and long-term (hypo)manic symptoms, anxiety, recurrence rates, rumination, dampening of positive affect, functional impairment, mindfulness skills, self-compassion, and positive mental health. Furthermore, potential moderators of treatment effects were examined.

## Method

### Study design

A multicentre, evaluator-blinded RCT comparing MBCT + TAU with TAU up to 15 months follow-up. Data were collected at seven outpatient clinics for adults with BD. The authors assert that all procedures contributing to this work comply with the ethical standards of the relevant national and institutional committees on human experimentation and with the Helsinki Declaration of 1975, as revised in 2008. All procedures involving participants were approved by the local ethics committee CMO Arnhem-Nijmegen for all participating sites (NL63319.091.17.). The study protocol has been published previously (Hanssen et al., 2019).

### Participants

Inclusion criteria were: (1) bipolar I or II disorder; (2) at least two confirmed lifetime depressive episodes; (3) at least one mood episode within the year prior to baseline; and (4) a baseline Young Mania Rating Scale (YMRS; Young, Biggs, Ziegler, & Meyer, 1978) score of ≤12. Exclusion criteria were: (1) insufficient comprehension of Dutch language; (2) previously participated in eight-week MBSR/MBCT; (3) severe manic episode within three months prior to baseline; (4) diagnosis of schizoaffective disorder, current substance abuse disorder, antisocial or borderline personality disorder; (5) increased risk of suicide or aggression; and (6) for practical reasons, were about to receive another psychological intervention between baseline and T1.

### Procedure

Participants were recruited at seven specialised outpatient clinics for adults with BD between May 2018 and February 2020. They were approached by their attending clinician. Those who were interested could contact the research team. Possible eligible participants were invited for a research interview with trained research assistants, where written informed consent was obtained from all participants, and in- and exclusion criteria were assessed and baseline assessment administered, after which participants were randomised to MBCT + TAU or TAU. Participants started MBCT within 1.5 months after randomisation. The baseline assessment (T0) and follow-up assessments at 3 (T1), 6 (T2), 9 (T3), 12 (T4), and 15 (T5) months after baseline consisted of self-report questionnaires and blinded clinician-administered assessments. Due to COVID-19 restrictions, clinician-administered assessments had to be scheduled by telephone from March 2020 onwards. There were no differences in primary outcome between interviews that were conducted face to face or by telephone at T1 (*t* = 0.18, *p* = 0.856) and T5 (*t* = −0.06, *p* = 0.954). At each time point participants were structurally asked whether they had experienced medical occurrences in order to reveal (serious) adverse events ((S)AEs) during the study period.

### Randomisation and blinding

Random assignment to MBCT + TAU or TAU (allocation ratio 1:1) was electronically conducted by Castor EDC (EDC, 2019). To control for possible confounding variables, randomisation was stratified by participating study sites, gender (Kuyken et al., 2010), bipolar subtype (type I or II), and depression status (current *v.* remitted) (van der Velden et al., 2022). Block randomisation with varying predefined blocks was used (2, 4, or 6). The coordinating researcher was blinded for block sizes; research assistants who conducted the research interviews were blinded for allocation. During the study period, it happened twice that a research assistant was unblinded. As there were multiple research assistants at every participating site, it was ensured that these participants were interviewed by another research assistant during the subsequent assessments.

### Intervention

#### Mindfulness-based cognitive therapy

The intervention was based on the original MBCT protocol for MDD (Segal, Williams, & Teasdale, 2012), with slight changes to tailor to BD, and consisted of eight weekly group sessions of 2.5 h and a six-hour day of guided silent practice. Participants were instructed to practice 45 min a day with guided formal (e.g. bodyscan, sitting meditation, movement exercises) and informal exercises (e.g. mindful routine activities, three-min breathing space). At each time point participants were asked whether they still practiced. See the study protocol for a complete overview of each MBCT session (Hanssen et al., 2019).

MBCT was taught by two teachers, of at least one fully qualified mindfulness teacher and one experienced in treating BD, in twelve groups with eight to ten participants (consisting of both study and non-study participants) on the respective study sites. See online Supplement 1 for more information on teacher competency. Due to COVID-19 restrictions, two MBCT groups were changed to an online videoconferencing format (from session 7 and session 4 respectively). Primary outcome from these two groups did not differ from groups who did not change to an online format (*t* = −1.10, *p* = 0.272).

#### Treatment as usual

Treatment as usual for participants with BD typically consists of pharmacotherapy, psychoeducation, and self-management interventions (Kupka et al., 2015). TAU was not restricted in any way to ensure clinical representativeness, except for asking participants not to start any psychological treatment between baseline and T1 for practical reasons. TAU, including medication use and other psychological treatments, was monitored by the Treatment Inventory of Costs in Patients with Psychiatric Disorders (TIC-P; Hakkaart, Van Straten, Donker, & Tiemens, 2002) during the entire follow-up period. Participants in the TAU group were offered MBCT after completing T5.

### Outcome measures

#### Primary outcome

The primary outcome measure was depressive symptoms at post-treatment, 3 months after baseline (T1), as assessed by trained research assistants using the 30-item *Inventory of Depressive Symptomatology – Clinician administered* (IDS-C; Akkerhuis, 1997). Internal consistency (IC) in the current study was good (Chronbach's *α* at T0 = 0.89). Audio recordings of IDS-C interviews were available for 20/22 research assistants. Of each assistant, two interviews were randomly selected and rated by two researcher psychologists (IH and MH) to determine interrater reliability. Intraclass correlation coefficient (ICC) estimates were calculated, based on a mean-rating (*k* = 2), absolute agreement, two-way random model with single measures. ICC was excellent (0.96, 95% CI [0.91–0.98]).

#### Secondary outcomes

The following clinician-administered assessments and self-report questionnaires were administered as secondary outcomes at each time point: The depression and mania module of the Structured Clinical Interview for DSM-IV Disorders (SCID-I; First, Spitzer, Gibbon, & Williams, 1996; First, Williams, Karg, & Spitzer, 2016); *Young Mania Rating Scale* (YMRS; Young et al., 1978, *α* = 0.52); *State/Trait Anxiety Inventory* (STAI; Van der Ploeg, 2000, *α* = 0.78 and *α* = 0.92 respectively); *Functioning Assessment Short Test* (FAST; Rosa et al., 2007, *α* = 0.85); *Mental Health Continuum – Short Form* (MHC-SF; Lamers, Westerhof, Bohlmeijer, ten Klooster, & Keyes, 2011, *α* = 0.92); 5-item *brooding subscale* of the *Ruminative Response Scale* – Extended version (RRS-br; Raes, Hermans, & Eelen, 2003, *α* = 0.79); *Responses to Positive Affect* (RPA; Feldman, Joormann, & Johnson, 2008, *α* = 0.86 and 0.78 respectively); *Five Facet Mindfulness Questionnaire – Short Form* (FFMQ-SF; Baer, Smith, Hopkins, Krietemeyer, & Toney, 2011, *α* = 0.86); *Self Compassion Scale – Short Form* (SCS-SF; Neff, 2003, *α* = 0.88); See the study protocol for more detailed information about these outcome measures (Hanssen et al., 2019).

### Statistical analyses

#### Sample size calculation

The power calculation was based on the estimated effect size of 0.5 on IDS-C scores at post-intervention (T1) (van Aalderen et al., 2012). Using an *α* of 0.05, a power of 80%, including a design factor of 1 − *r*^2^ (0.75) (Borm, Fransen, & Lemmens, 2007), and taking into account a conservative estimate of 40% loss to follow-up at T1, 80 participants per group were required, a total of 160 participants (Hanssen et al., 2019). Towards the end of the inclusion period, due to COVID-19 restrictions, we had to prematurely end recruitment of the study at *N* = 144. After consultation of the Ethical Committee, an additional power analysis with the actual loss to follow-up at T1 (20% rather than 40%) was conducted, revealing that a total number of 120 participants would have sufficed.

#### Treatment effects at T1

All analyses were conducted in SPSS version 25 (IBM Corp., 2017) and performed on both the intention-to-treat (ITT) sample, consisting of all randomised participants, and the per protocol (PP) sample, consisting of participants who adhered to the treatment protocols (MBCT + TAU group: attended ≥4 MBCT sessions; and TAU group: did not attend a mindfulness-based intervention). The percentage of missing data for our primary outcome at post-treatment was 18.1% in MBCT + TAU and 20.8% in TAU. Participants for whom the primary outcome was missing did not differ from the others on any of the baseline measures. Therefore, we assumed these data to be missing completely at random (MCAR). Visual inspection of histograms did not reveal any strong violations of skewness or kurtosis, therefore no transformations were used. Post-treatment analyses were conducted with Linear Mixed Effect models, with time, group (MBCT + TAU/TAU) and their interaction added as fixed effects, while controlling for stratification variables (i.e. study site, gender, type of BD, and depression status). A random intercept for participants was added. A diagonal covariance structure was used. Restricted maximum likelihood was used as estimation method to handle missing data. Due to the small number of participants per MBCT group, we no longer clustered for treatment group as described in our study protocol (Hanssen et al., 2019). The intra-cluster correlation coefficient (ICC) was calculated in order to investigate the amount of variance in the primary outcome measure (IDS-C at T1) that could be explained by treatment group (ICC = treatment group variance / treatment group variance + residual variance). This resulted in an ICC of 0.2. Cohen's *d* effect size was calculated by dividing the adjusted group difference at T1 by the pooled standard deviation at T0. Due to the absence of a treatment effect, the planned mediation analyses as described in our study protocol were not conducted (Hanssen et al., 2019).

#### Follow-up effects

The consolidation of treatment effects over the follow-up period for both primary and secondary outcomes at T1, T2, T3, T4, and T5 were evaluated with Linear Mixed Effects Models, including the same parameters as mentioned above, while controlling for baseline levels and stratification variables. A heterogeneous first-order autoregressive [ARH(1)] covariance structure was used. Restricted maximum likelihood was used as estimation method to handle missing data. Cohen's *d* was calculated by dividing the adjusted group difference between the pooled means (T1, T2, T3, T4, T5) by the pooled standard deviation at T0.

#### Recurrence

Participants with a depressive or (hypo)manic episode at baseline were excluded from analysis. Number of recurrences and severity of first episode based on number of DSM-5 symptoms (depression: 5 = mild, 6/7 = moderate and 8/9 = severe; (hypo)mania: 3 = mild, 4/5 = moderate and 6/7 = severe) were compared with a Pearson χ^2^-test. Visual inspection of histograms of the continuous variables (e.g. duration of first episodes, total number of days depressed/(hypo)manic) revealed violations of skewness and kurtosis, therefore the Mann–Whitney *U* test was used as non-parametric analysis.

Time to recurrence was calculated in weeks from the start of the study until the first recurrence. Differences in time to recurrence were analysed using a Cox regression proportional hazards model. Analyses were performed with adjustment for the number of previous depressive and (hypo)manic episodes, baseline levels of depressive and (hypo) manic symptoms (Altman et al., 2006), and stratification variables. The proportional hazards function assumption was not violated. Participants with missing data and those who did not experience a recurrence within the 15 months follow-up were treated as censored observations. Participants who discontinued the trial due to mood episodes were included in the analysis. The last observation data was used for participants who discontinued the trial for different reasons (e.g. having no time).

#### Moderation analyses

Moderation analyses controlling for baseline depressive symptoms were performed by adding potential predictors and its interaction with group to above mentioned models. Separate models were run for each possible moderator. The following possible moderators were used: gender, age, type of BD, total number of life-time mood episodes (<12 or ≥12), age of onset, polarity (based on number of life-time depressive and (hypo)manic episodes), baseline levels of depressive symptoms (IDS-C), rumination (RRS-br), and functional impairment (FAST), and childhood trauma (measured with the Childhood Trauma Questionnaire (CTQ; Bernstein et al., 2003).

## Results

### Sample characteristics

**Fig. 1.**
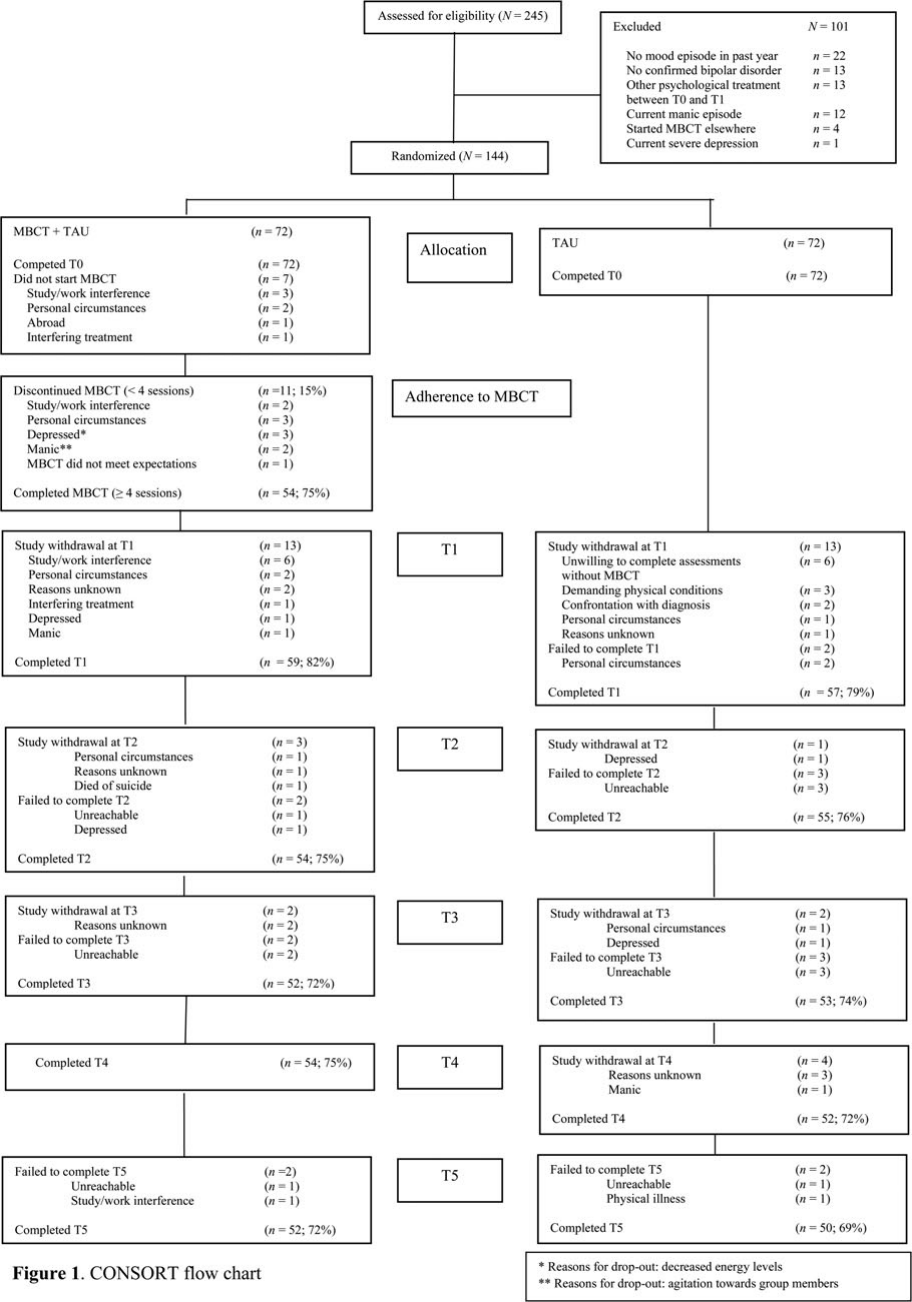
CONSORT flow chart.

In total, 144 participants were randomly allocated to MBCT + TAU (*n* = 72) and TAU (*n* = 72) (Fig. 1). Participants were mostly female (60%) and highly educated (56%). Mean age was 46.6 (s.d. = 12.7). See online Supplement 2 for the baseline sociodemographic and clinical characteristics. Within the MBCT + TAU group, participants who attended <4 MBCT sessions (*n* = 18; 25%) did not differ from those who completed MBCT. Within the MBCT + TAU group, 51 (71%) participants indicated they still practiced formal mindfulness exercises regularly at T1 [mean frequency per week = 3.5 (s.d. = 5.1) and mean minutes per week = 21.0 (s.d. = 15.0)]. At T2, this number declined to 37 (51%) participants (mean frequency per week = 2.7 (s.d. = 2.5) and mean minutes per week = 15.2 (s.d. = 14.8) and this remained as such until T5.

Between T0 and T1 more participants in TAU visited a mental health care professional than in MBCT + TAU (χ^2^ = 3.0, *p* = 0.049) and between T1 and T2 participants in TAU had more frequent mental health care professional visits than those in MBCT + TAU (*t* = 2.1, *p* = 0.035) (online Supplement 3).

### Treatment effects at T1

ITT analysis revealed no significant group differences in depressive symptoms (Table 1). MBCT + TAU showed a significant increase in mindfulness skills (*d* = 0.32) compared to TAU. No significant group differences were found in other secondary outcome measures. PP analyses revealed similar results.

**Table 1.** Intention to treat analyses on primary and secondary outcomes at post-treatment

		MBCT + TAU (*n* = 72)	TAU (*n* = 72)				Results linear mixed models		
		Unadjusted mean M (S.D.)		Adjusted Group difference [95% CI]	*d* [95% CI]		*F*	df	*p*
Primary outcome									
Depressive symptoms (IDS-C)	T0	15.2 (13.4)	15.8 (11.8)			Study site	1.5	6.126	0.197
	T1	13.7 (9.2)	16.3 (12.0)	−2.6 [−7.0 to 1.8]	0.24 [−0.1 to 0.6]	Group	1.2	1.118	0.268
						Time	0.1	1.118	0.804
						Group × Time	1.1	1.118	0.303
Secondary outcomes									
(Hypo)manic symptoms (YMRS)	T0	2.1 (2.6)	1.9 (2.5)			Study site	1.1	6.122	0.350
	T1	2.2 (3.3)	2.3 (3.2)	−0.3 [−1.5 to 0.8]	0.12 [−0.2 to 0.5]	Group	0.0	1.113	0.851
						Time	0.8	1.117	0.379
						Group × Time	0.6	1.117	0.447
Anxiety state symptoms (STAI-state)	T0	43.6 (7.5)	44.4 (6.8)			Study site	0.8	6.126	0.560
	T1	42.8 (6.8)	44.7 (7.4)	−2.2 [−0.5 to 4.8]	0.31 [−0.1 to 0.7]	Group	2.0	1.124	0.159
						Time	0.0	1.112	0.850
						Group × Time	0.9	1.112	0.344
Anxiety trait symptoms (STAI-trait)	T0	45.4 (10.4)	49.0 (10.4)			Study site	1.2	6.127	0.304
	T1	42.4 (9.8)	46.8 (9.9)	−5.1 [−8.5 to −1.7]	0.49 [0.1–0.9]	Group	7.7	1.127	0.006
						Time	15.4	1.109	<0.001
						Group × Time	1.4	1.109	0.238
Functional impairment (FAST)	T0	14.9 (11.1)	15.4 (10.5)			Study site	1.6	6.129	0.157
	T1	12.8 (10.9)	16.6 (11.6)	−3.8 [−7.5 to 0.1]	0.44 [0.1–0.8]	Group	2.2	1.126	0.141
						Time	0.0	1.115	0.859
						Group × Time	2.9	1.115	0.087
Positive Mental Health (MHC)	T0	2.6 (1.0)	2.3 (1.1)			Study site	0.5	6.128	0.844
	T1	2.7 (1.0)	2.5 (1.1)	0.3 [−0.7 to 0.1]	0.29 [−0.1 to 0.7]	Group	2.4	1.127	0.120
						Time	3.1	1.109	0.079
						Group × Time	0.0	1.109	0.831
Rumination NA (RRS-br)	T0	11.2 (3.2)	11.8 (3.3)			Study site	1.7	6.122	0.127
	T1	9.5 (2.7)	10.4 (2.6)	−1.2 [−2.2 to −0.2]	0.16 [−0.2 to 0.5]	Group	4.1	1.124	0.046
						Time	33.8	1.117	<0.001
						Group × Time	1.0	1.117	0.312
Dampening PA (RPA)	T0	14.8 (4.4)	15.0 (3.9)			Study site	2.4	6.124	0.032
	T1	13.2 (3.9)	14.6 (4.0)	−1.5 [−3.0 to −0.1]	0.36 [0.0–0.7]	Group	2.3	1.124	0.135
						Time	5.9	1.111	0.016
						Group × Time	3.3	1.111	0.071
Rumination PA (RPA)	T0	22.4 (5.1)	20.2 (5.1)			Study site	0.4	6.123	0.903
	T1	22.9 (5.1)	20.6 (5.2)	1.8 [−3.8 to 0.2]	0.35 [0.0–0.7]	Group	5.2	1.119	0.025
						Time	0.5	1.108	0.474
						Group × Time	0.1	1.108	0.804
Mindfulness skills (FFMQ)	T0	79.7 (12.1)	76.5 (11.9)			Study site	1.0	6.127	0.459
	T1	83.9 (11.4)	78.6 (10.0)	6.2 [−10.1 to −2.4]	0.58 [0.2–0.9]	Group	6.4	1.127	0.012
						Time	15.9	1.113	<0.001
						**Group** × **Time**	**4**.**2**	**1.114**	**0**.**041**
Self-compassion (SCS)	T0	3.8 (1.1)	3.6 (1.0)			Study site	0.8	6.128	0.612
	T1	4.1 (1.1)	3.9 (1.1)	0.4 [−0.0 to 0.0]	0.38 [0.0–0.7]	Group	2.5	1.128	0.113
						Time	12.1	1.112	0.001
						Group × Time	0.9	1.112	0.344

Note: IDS-C, Inventory of Depressive Symptomatology – Clinician rated; YMRS, Young Mania Rating Scale; STAI, State-trait Anxiety Inventory; FAST, Functioning Assessment Short Test; MHC, Mental Health Continuum; RRS-br, Ruminative Response Scale – brooding subscale; RPA, Responses to Positive Affect; FFMQ, Five Facet Mindfulness Questionnaire; SCS, Self Compassion Scale

Bold means that there is a significant group × time interaction.

### Effects at follow-up

ITT analyses revealed that there were no significant differences between groups over the course of the follow-up regarding depressive symptoms (Table 2). Significant time x group interactions were found on state and trait anxiety, mindfulness skills, and positive mental health. No significant time x group interactions were found on other secondary outcome measures. PP analyses revealed similar results.

**Table 2.** Intention to treat analyses on primary and secondary outcomes at follow-up

	MBCT + TAU (*n* = 72)	TAU (*n* = 72)				Results linear mixed models		
	Unadjusted mean M (S.D.)		Adjusted Group difference [95% CI]	*d* [95% CI]		*F*	df	*p*
Primary outcome								
Depressive symptoms (IDS-C)			−1.59 [−2.2 to 6.3]	0.13 [−0.2 to 0.5]	Study site	2.3	6.100	0.037
T0	15.2 (13.4)	15.8 (11.8)			Group	2.0	1.103	0.157
T1	13.8 (9.2)	16.3 (12.0)			Time	2.4	4.150	0.050
T2	12.6 (10.3)	16.0 (11.8)			Group × Time	0.5	4.150	0.724
T3	12.8 (10.9)	15.0 (13.3)						
T4	12.9 (11.5)	14.0 (12.7)						
T5	11.4 (10.1)	11.2 (11.2)						
Secondary outcomes								
(Hypo)manic symptoms (YMRS)			0.1 [−0.8 to 1.0]	0.04 [−0.4 to 0.3]	Study site	1.1	6.92	0.392
T0	2.1 (2.6)	1.9 (2.5)			Group	0.3	1.93	0.589
T1	2.2 (3.3)	2.3 (3.2)			Time	0.8	4.127	0.549
T2	1.8 (2.7)	1.8 (2.2)			Group × Time	0.2	4.127	0.921
T3	1.8 (2.6)	1.6 (2.1)						
T4	1.5 (2.7)	2.0 (2.9)						
T5	1.7 (3.1)	1.7 (2.6)						
Anxiety state symptoms (STAI-state)					Study site	1.8	6.93	0.097
T0	43.6 (7.5)	44.4 (6.8)			Group	0.0	1.91	0.875
T1	42.8 (6.8)	44.7 (7.4)	−1.9 [−4.2 to 0.5]	0.27 [−0.1 to 0.6]	Time	3.7	4.118	0.007
T2	41.7 (6.9)	42.4 (6.2)	−0.8 [−3.3 to 1.6]	0.10 [−0.3 to 0.5]	**Group** × **Time**	**4**.**0**	**4.118**	**0**.**005**
T3	41.6 (6.6)	42.3 (7.5)	−1.1 [−3.7 to 1.6]	0.10 [−0.3 to 0.5]				
T4	42.2 (7.7)	38.8 (6.3)	3.0 [0.5–5.5]	0.48 [−0.4 to −0.1]				
T5	42.1 (7.3)	40.3 (5.7)	1.5 [−0.9 to 3.8]	0.25 [−0.6 to 0.1]				
Anxiety trait symptoms (STAI-trait)					Study site	1.1	6.91	0.357
T0	45.4 (10.4)	49.0 (10.4)			Group	0.0	1.103	0.900
T1	42.4 (9.8)	46.8 (9.9)	−2.7 [−5.2 to −0.2]	0.42 [0.1–0.8]	Time	1.9	4.112	0.108
T2	41.6 (10.1)	45.2 (9.8)	−1.3 [−4.0 to 1.5]	0.35 [0.0–0.7]	**Group** × **Time**	**3**.**9**	**4.112**	**0**.**005**
T3	41.8 (10.2)	44.7 (11.4)	−1.1 [−4.5 to 2.4]	0.28 [−0.1 to 0.6]				
T4	42.7 (10.7)	41.3 (10.3)	1.9 [−1.2 to 4.9]	0.14 [−0.5 to 0.2]				
T5	42.7 (11.5)	41.9 (10.5)	2.5 [−0.7 to 5.6]	0.08 [−0.4 to 0.3]				
Functional impairment (FAST)			−2.5 [−1.3 to 6.4]	0.23 [−0.1 to 0.6]	Study site	2.2	6.99	0.046
T0	14.9 (11.1)	15.4 (10.5)			Group	3.4	1.102	0.069
T1	12.8 (10.9)	16.6 (11.6)			Time	6.5	4.177	<0.001
T2	10.4 (9.8)	14.7 (10.9)			Group × Time	2.4	4.177	0.052
T3	10.9 (10.6)	14.1 (11.1)						
T4	9.9 (10.6)	13.0 (11.2)						
T5	9.8 (8.8)	9.2 (9.3)						
Positive Mental Health (MHC)					Study site	1.2	6.94	0.313
T0	2.6 (1.0)	2.3 (1.1)			Group	1.4	1.94	0.243
T1	2.7 (1.0)	2.5 (1.1)	0.1 [−0.2 to 0.4]	0.19 [−0.6 to 0.2]	Time	2.1	4.116	0.088
T2	2.8 (1.0)	2.7 (1.1)	0.0 [−0.3 to 0.3]	0.09 [−0.5 to 0.3]	**Group** × **Time**	**5**.**0**	**4.117**	**0**.**001**
T3	2.7 (0.9)	2.7 (1.2)	−0.1 [−0.4 to 0.3]	0.00 [−0.4 to 0.4]				
T4	2.6 (0.9)	2.9 (1.0)	−0.3 [−0.6 to 0.1]	0.29 [−0.1 to 0.7]				
T5	2.6 (1.1)	3.1 (1.1)	−0.5 [−0.8 to −0.2]	0.48 [0.1–0.8]				
Rumination NA (RRS-br)			−0.5 [−0.3 to 0.5]	0.15 [−0.2 to 0.5]	Study site	2.8	6.96	0.016
T0	11.2 (3.2)	11.8 (3.3)			Group	1.7	1.98	0.199
T1	9.5 (2.7)	10.4 (2.6)			Time	2.3	4.116	0.062
T2	8.9 (2.4)	9.9 (2.6)			Group × Time	2.2	4.116	0.070
T3	9.0 (2.8)	10.1 (3.0)						
T4	9.3 (3.3)	9.0 (2.8)						
T5	9.3 (2.8)	9.0 (3.1)						
Dampening PA (RPA)			−0.9 [−0.5 to 2.2]	0.20 [−0.2 to 0.6]	Study site	2.2	6.97	0.052
T0	14.8 (4.4)	15.0 (3.9)			Group	3.1	1.97	0.083
T1	13.2 (3.9)	14.6 (4.0)			Time	1.1	4.116	0.340
T2	13.3 (3.6)	14.2 (3.6)			Group × Time	1.0	4.116	0.407
T3	13.1 (3.4)	13.6 (3.6)						
T4	12.8 (3.1)	13.3 (3.4)						
T5	12.9 (3.4)	13.5 (4.2)						
Rumination PA (RPA)			0.5 [−1.4 to 2.4]	0.10 [−0.5 to 0.3]	Study site	0.9	6.99	0.518
T0	22.4 (5.1)	20.2 (5.1)			Group	0.3	1.100	0.560
T1	22.9 (5.1)	20.6 (5.2)			Time	0.5	4.112	0.749
T2	22.7 (5.1)	20.7 (5.3)			Group × Time	1.0	4.113	0.396
T3	22.6 (5.4)	19.8 (4.6)						
T4	21.8 (5.3)	20.9 (4.9)						
T5	22.0 (5.3)	21.1 (5.6)						
Mindfulness skills (FFMQ)					Study site	0.1	6.89	0.988
T0	79.7 (12.1)	76.5 (11.9)			Group	0.1	1.105	0.703
T1	83.9 (11.4)	78.6 (10.0)	4.4 [1.4–7.4]	0.44 [−0.8 to −0.1]	Time	3.5	4.112	0.010
T2	84.7 (13.1)	81.9 (11.7)	1.4 [−2.2 to 4.9]	0.23 [−0.6 to 0.1]	**Group** × **Time**	**5**.**5**	**4.112**	**<0**.**001**
T3	82.7 (13.4)	81.0 (10.7)	0.7 [−3.0 to 4.4]	0.14 [−0.5 to 0.2]				
T4	84.2 (12.8)	84.3 (12.1)	−0.7 [−4.4 to 3.0]	0.01 [−0.4 to 0.4]				
T5	83.5 (13.2)	85.7 (12.3)	−3.2 [−6.8 to 0.5]	0.18 [−0.2 to 0.5]				
Self-compassion (SCS)			0.1 [−0.3 to 0.5]	0.09 [−0.5 to 0.3]	Study site	0.4	6.99	0.851
T0	3.8 (1.1)	3.6 (1.0)			Group	0.4	1.99	0.526
T1	4.1 (1.1)	3.9 (1.1)			Time	3.6	4.104	0.008
T2	4.4 (1.1)	4.1 (1.0)			Group × Time	2.0	4.104	0.105
T3	4.3 (1.1)	4.0 (1.1)						
T4	4.2 (1.1)	4.4 (1.1)						
T5	4.3 (1.2)	4.3 (1.1)						

Note: IDS-C, Inventory of Depressive Symptomatology – Clinician rated; YMRS, Young Mania Rating Scale; STAI, State-trait Anxiety Inventory; FAST, Functioning Assessment Short Test; MHC, Mental Health Continuum; RRS-br, Ruminative Response Scale – brooding subscale; RPA, Responses to Positive Affect; FFMQ, Five Facet Mindfulness Questionnaire; SCS, Self Compassion Scale

Bold means that there is a significant group × time interaction.

#### Recurrence

ITT analyses revealed no differences in recurrence between MBCT + TAU and TAU. In total, 32 (49%) participants in MBCT + TAU and 35 (55%) participants in TAU developed recurrence in a mood episode over the 15-month follow-up period. Of these, 18 (28%) in MBCT + TAU and 22 (34%) in TAU showed recurrence in a depressive episode first, while 14 (22%) in MBCT + TAU and 13 (20%) in TAU showed recurrence in a (hypo)manic episode (χ^2^ = 0.585, *p* = 0.747). PP analyses showed similar results. In MBCT + TAU, the duration of the first depressive recurrence in days was shorter (Mdn = 21.5) than in TAU (Mdn = 61; U = 114.0, *p* = 0.035). ITT analyses revealed no differences between MBCT + TAU and TAU in number of recurrences, total days depressed or (hypo)manic, severity of first depressive or (hypo)manic recurrence, or duration of first (hypo)manic recurrence in days. Online Supplements 6 and 7 show the survival curves of non-recurrence in respectively depression and (hypo)mania over the 15 months follow-up period. No differences were found in recurrence in depression (hazard ratio = 0.70, 95% CI [0.21–2.38], *p* = 0.570) or (hypo)mania (hazard ratio = 1.05, 95% CI [0.19–5.88], *p* = 0.953) between MBCT + TAU and TAU. PP analyses showed similar results.

### Moderation analyses

Participants with higher depressive symptom severity at baseline had less depressive symptoms at T1 in MBCT + TAU compared to TAU(*F* (1112) = 11.161, 95% CI [0.19–0.77], *d* = 0.56, *p* = 0.001; See online Supplement 6). Furthermore, participants with higher functional impairment at baseline had less depressive symptoms at T1 in MBCT + TAU compared to TAU (*F* (1111) = 4.204, 95% CI [0.01–0.65], *d* = 0.34 *p* = 0.043; see online Supplement 7). No other moderators of treatment outcome were found. Similar results were found over the course of 15-month follow-up and in the PP sample.

### (Serious) adverse events

During follow-up, three participants in MBCT + TAU and three participants in TAU experienced SAEs. In the MBCT + TAU group, these SAEs included: heart attack (*n* = 1), intoxication with medicine (*n* = 1), and suicide (*n* = 1). In the TAU group, the SAEs included: surgery (*n* = 2, oesophagus and unknown), and clinical admission due to severe depressive episode (*n* = 1). After consideration by the medical-ethical committee, all SAEs were deemed unrelated to the study or MBCT. In total, 25 (35%) participants in MBCT + TAU and 20 (28%) participants in TAU reported AEs. These AEs were divided into two main categories, namely somatic illness / physical pain (e.g. coronavirus, flu, migraine) (MBCT + TAU: *n* = 23; TAU: *n* = 17), and side effects from medication (MBCT + TAU: *n* = 4; TAU: *n* = 6). See Hanssen et al. (2021a) for a complete overview of reported difficult experiences during MBCT.

## Discussion

### Main findings

The current RCT showed that MBCT + TAU was not more efficacious than TAU in reducing current depressive symptoms in the whole sample. However, exploratory moderation analyses showed that participants with higher depressive symptoms and more functional impairment at baseline benefitted from MBCT in terms of their improvement of depressive symptoms, both at post-treatment and during the 15 month follow-up period. During follow-up, participants in the TAU condition showed more positive mental health and less anxiety than those receiving MBCT + TAU. At post-treatment, MBCT + TAU showed higher levels of mindfulness skills. However, during follow-up, TAU showed a stronger increase in mindfulness skills than MBCT + TAU. Perhaps participants in the TAU condition engaged in mindfulness as well, for example, by the use of meditation apps, which could explain their improvement. There were no other differences between groups.

Our finding that MBCT on average did not reduce depressive symptoms is in contrast with previous studies on efficacy of MBCT in unipolar depression (Kuyken et al., 2016). An explanation might be that participants in the current study showed relatively mild depressive symptoms at baseline, leaving little space for improvement. Indeed, exploratory moderation analyses revealed that participants with higher depressive symptom levels at baseline benefitted more from MBCT in comparison with TAU. This is in line with a study on an online mindfulness-based intervention in BD (Murray et al., 2021) and an online self-management intervention in BD (Gliddon et al., 2019). Based on these findings, current depressive symptoms do therefore not have to be an exclusion criterium to participate in an intensive psychological treatment such as MBCT. Furthermore, MBCT did not appear to reduce the risk of depressive recurrence at follow-up, which is in contrast with previous studies in unipolar depression (Kuyken et al., 2016), but in line with other RCTs in people with BD (de Dios et al., 2021; Perich et al., 2013). As there was a loss to follow-up and participants with current mood episodes at T0 were excluded from the recurrence analysis, the resulting power might have been insufficient to detect this. Furthermore, given the high recurrence risk in BD despite various evidence-based treatment options, it might also be important to focus on the severity and duration of episodes rather than just recurrence risk. Our findings show that MBCT decreased the duration of the first depressive recurrence, which might be an important indicator that MBCT could have a protective effect in overall impact of recurrence. Furthermore, people with BD can suffer from persisting alterations in psychosocial functioning up to one year after remission (Rosa et al., 2010). This emphasises the need for psychological interventions targeted at improving functional outcomes in this group. Our exploratory findings show that participants with higher functional impairment at baseline benefitted more from MBCT. As this is the first RCT that shows possible beneficial effects of MBCT on functioning in BD, it is important to replicate these findings.

### Limitations

There are several limitations that should be mentioned. First, we have no data of participants who declined to participate. Second, the COVID-19 pandemic required some adaptations to our study. We stopped recruitment early, two MBCT groups had to change to an online format, and follow-up assessments had to be conducted by telephone. However, as there were no differences between these groups on outcomes, we expect that the impact of these changes was minimal. Third, even though we included a 15 months follow-up period, this still might be too short to capture the effect of an intervention. Longer study duration might be necessary to capture the possible effects of mindfulness on the pattern of mood episodes in the long run. Fourth, the sample size might not be large enough to fit adequate statistical models. Therefore, results from the moderation analysis should be considered preliminary. Fifth, perhaps the limited effectiveness of the current study could be due to the fact that more than 50% of our participants were taught by mindfulness teachers that were classified as ‘beginner’ or ‘advanced beginner’. This was also related to a paucity of mindfulness teachers in the expertise centres for BD s, which also reflects current clinical practice.

### Research implications

This study indicates that MBCT is not beneficial for all people with BD, but that it may be particularly helpful for people with higher depressive symptoms and functional impairment at baseline. In addition, in contrast with Scott et al. (2006) who found that CBT appeared to be less effective in people with BD who experienced more than 12 mood episodes, we did not find that MBCT was less effective in this group. For the latter category of people with BD in particular, MBCT might fill a gap in available efficacious psychotherapeutic interventions. As only a small number of participants were moderately to severely depressed or functionally impaired in the current study, it is important that future research replicates these findings, for example by including a cut-off for depressive symptom severity. Determining the efficacy of MBCT in subgroups with certain profiles could help to address the need for personalised treatment options in BD. This would resonate with findings in a recent network meta-analysis showing that adjunctive psychotherapy is the best option in stabilising episodes and preventing recurrences in BD, while at the same time heterogeneity in study populations was found to temper this finding (Miklowitz et al., 2021). Furthermore, as participants in our qualitative study indicated that they experienced the lack of follow-up sessions as a barrier to maintain their meditation practice (Hanssen et al., 2021b), future research should investigate whether including booster sessions after MBCT increases long-term adherence and effectiveness. Moreover, the use of ecological momentarily assessments by interviews and self-report questionnaires in a highly recurrent and alternating disorders such as BD might be questionable. Experience sampling methods (ESM) might give a more adequate representation of the effect of an intervention on daily life symptom experience (Verhagen, Hasmi, Drukker, van Os, & Delespaul, 2016). It might be worthwhile to include ESM measures in future studies on MBCT in BD, especially as this is a group whose daily lives are often severely disrupted by mood symptoms and alterations in psychosocial functioning. This could also provide the opportunity to not only measure a count of relapses, but instead to measure the severity and duration of relapse as well. Moreover, it is important for future studies to not only include outcome measures that focus on clinical recovery, but also on functional, social, and personal recovery, such as quality of life. Future research should therefore not only be directed to the question if MBCT is effective in people with BD, but also in what stage of the disorder they might benefit most. Finally, future mediation, moderation, neuroimaging, and cognitive experimental studies might be helpful in identifying specific individual profiles and working mechanisms of MBCT in people with BD. This will be helpful in optimising MBCT for BD and facilitating the decision making process of successful implementation of MBCT in treatment guidelines of BD.

### Future directions

The current findings suggest that MBCT could be safely delivered to people with BD with current depressive symptoms and functional impairment. However, the current state of evidence is not sufficient to be able to include MBCT in current treatment guidelines. Future research is necessary to be able to determine whether or when MBCT might be most effective in people with BD.

## Data Availability

Data will be made available in a public data respiratory upon publication: Data Archiving and Networked Services (DANS).

## Supplementary material

For supplementary material accompanying this paper visit https://doi.org/10.1017/S0033291723000090. click here to view supplementary material
