# Supplementary material for: Mindfulness-based cognitive therapy v. treatment as usual in people with bipolar disorder: A multicentre, randomised controlled trial
Source: Psychol Med. 2023 Mar 7;53(14):6678–90. doi: 10.1017/S0033291723000090 (PMC10600813; doi:10.1017/S0033291723000090)
Supplement: Supplementary file 1 [file S0033291723000090sup.zip › S0033291723000090sup001.docx]

**Supplement 1 – Teacher competency**

MBCT groups were taught by two teachers, at least one of whom was fully qualified according to the criteria of the UK Network for Mindfulness-Based Teachers (Crane, Soulsby, Kuyken, Williams, & Eames, 2011), and one of whom was experienced in treating BD. All teachers received two supervision sessions with AS, professor of psychiatry and experienced mindfulness teacher. Teacher competence and adherence to the protocol were assessed by the Mindfulness-Based Interventions – Teacher Assessment Criteria (MBI-TAC; Crane et al., 2011). Videotaped sessions were available for 5/7 mindfulness teachers. Videotapes of two teachers were missing due to technical issues. There were no differences in outcomes between groups of videotaped teachers and the two groups which were not videotaped (t = -0.43, p = 0.667). From each teacher two sessions were randomly selected and rated independently by two experienced mindfulness teachers. Based at competency levels of the assessed teachers, 21 (29%) patients were taught by teachers classified as competent, 25 (35%) by advanced beginners, and 9 (13%) by beginners.
