# Supplementary material for: Mindfulness-based cognitive therapy v. treatment as usual in people with bipolar disorder: A multicentre, randomised controlled trial
Source: Psychol Med. 2023 Mar 7;53(14):6678–90. doi: 10.1017/S0033291723000090 (PMC10600813; doi:10.1017/S0033291723000090)
Supplement: Supplementary file 1 [file S0033291723000090sup.zip › S0033291723000090sup002.docx]

**Supplement 2. Baseline sociodemographic and clinical characteristics**

| Supplement 2. Baseline sociodemographic and clinical characteristics | | | |
| --- | --- | --- | --- |
|  | **Total**  **(*N* = 144)** | **MBCT + TAU**  **(*n* = 72)** | **TAU**  **(*n* = 72)** |
| Demographic characteristics | *n (%)* | *n (%)* | *n (%)* |
| Female gender | 87 (60) | 41 (57) | 46 (64) |
| Age; M (S.D.) | 46.6 (12.7) | 47.1 (12.1) | 46.1 (13.3) |
| Married/living together | 66 (46) | 32 (44) | 34 (47) |
| Employment status  Employed  Unemployed  Volunteer  Other (student/retired) | 48 (33)  53 (37)  21 (15)  18 (13) | 30 (42)  23 (32)  8 (11)  9 (13) | 18 (25)  30 (42)  13 (18)  9 (13) |
| Educational level ^c^  Low  Middle High | 14 (10)  45 (31)  80 (56) | 8 (11)  23 (32)  39 (54) | 6 (8)  22 (31)  42 58) |
|  |  |  |  |
| Clinical characteristics | ***n (%)*** | ***n (%)*** | ***n (%)*** |
| Subtype of BD, DSM-5  BD type I BD type II | 86 (60)  58 (40) | 43 (60)  29 (40) | 43 (60)  29 (40) |
| Age of onset; M (S.D.) | 23.2 (10.2) | 24.2 (10.8) | 22.1 (9.5) |
| Lifetime initial episode  Depressive  (Hypo)manic  Mixed | 89 (62)  49 (34)  4 (3) | 46 (64)  25 (35)  1 (1) | 43 (60)  24 (33)  5 (7) |
| Lifetime polarity  Depressive polarity  (Hypo)manic polarity  No polarity | 65 (45)  17 (12)  62 (43) | 33 (46)  8 (11)  31 (43) | 32 (44)  9 (13)  31 (43) |
| Lifetime number of episodes; M (SD)  Depressive  (Hypo)manic | 20.2 (31.2)  11.7 (25.9) | 19.4 (25.0)  10.7 (15.1) | 21.0 (36.6)  12.7 (33.4) |
| Comorbidity, DSM-5  (Mild) Alcohol Use Disorder  (Mild) Substance Use Disorder  Panic disorder  Social Anxiety Disorder  Specific Phobia  Generalized Anxiety Disorder  Obsessive Compulsive Disorder  Posttraumatic Stress Disorder | 1 (1)  2 (1)  14 (10)  10 (7)  9 (6)  10 (7)  2 (1)  9 (6) | 1 (1)  0 (0)  6 (8)  3 (4)  3 (4)  4 (5)  1 (1)  4 (6) | 0 (0)  2 (3)  8 (11)  7 (10)  6 (8)  6 (8)  1 (1)  5 (7) |
| Current use of mood stabilizing medication  Mood stabilizer Antidepressants Antipsychotics | 108 (75)  33 (23)  41 (28) | 66 (92)  55 (76)  15 (21)  15 (21) | 65 (90)  53 (74)  18 (25)  26 (36) |
| Previous psychoeducation | 111 (77) | 54 (75) | 57 (79) |
| Previous psychological treatment  Cognitive Behavioral Therapy  Family Systems Therapy  Schema Therapy  Other | 90 (63)  18 (13)  19 (13)  51 (35) | 43 (60)  14 (19)  12 (17)  28 (39) | 47 (65)  4 (6)  7 (10)  23 (32) |
| Outcome measures | *M* (S.D.) | *M* (S.D.) | *M* (S.D.) |
| Depressive symptoms (IDS-C) | 15.5 (12.6) | 15.2 (11.8) | 15.8 (11.8) |
| (Hypo)Manic symptoms (YMRS) | 2.0 (2.6) | 2.1 (2.6) | 1.9 (2.5) |
| STAI state^d^ | 44.0 (7.1) | 43.6 (7.5) | 44.4 (6.8) |
| STAI trait^d^ | 47.2 (10.5) | 45.4 (10.4) | 49.0 (10.4) |
| Rumination (RRS-br) | 11.5 (3.3) | 11.2 (3.2) | 11.8 (3.3) |
| Response to positive affect (RPA)  Dampening  Rumination | 14.9 (4.2)  21.3 (5.2) | 14.8 (4.4)  22.4 (5.1) | 15.0 (3.9)  20.2 (5.1) |
| Functional impairment (FAST) | 15.2 (10.8) | 14.9 (11.1) | 15.4 (10.5) |
| Mindfulness skills (FFMQ-SF) | 78.1 (12.0) | 79.7 (12.1) | 76.5 (11.9) |
| Self-compassion (SCS-SF) | 3.7 (1.1) | 3.8 (1.1) | 3.6 (1.0) |
| Positive mental health (MHC-SF) | 2.4 (1.0) | 2.6 (1.0) | 2.3 (1.1) |
