# Supplementary material for: Mindfulness-based cognitive therapy v. treatment as usual in people with bipolar disorder: A multicentre, randomised controlled trial
Source: Psychol Med. 2023 Mar 7;53(14):6678–90. doi: 10.1017/S0033291723000090 (PMC10600813; doi:10.1017/S0033291723000090)
Supplement: Supplementary file 1 [file S0033291723000090sup.zip › S0033291723000090sup003.docx]

| **Supplement 3. Mental health care utilization TAU** | | | | |
| --- | --- | --- | --- | --- |
|  | **MBCT+TAU**  **(*n* = 72)** | **TAU**  **(*n* = 72)** |  |  |
| **Percentage of patients using mood stabilizing medication between time points* / **** | | | | |
|  | ***n (*%)** | ***n (*%)** | **χ^2^** | ***p*** |
| T0 – T1  Stable medication  Started medication  Stopped medication | *n = 58*  53 (91)  1 (2)  4 (7) | *n* = 53  51 (96)  2 (4)  0 (0) | 1.1  0.5  3.7 | 0.441  0.603  0.120 |
| T1 – T2  Stable medication  Started medication  Stopped medication | *n* = 50  45 (90)  3 (6)  2 (4) | *n* = 49  49 (100)  0 (0)  0 (0) | 0.5  3.1  2.0 | 0.484  0.245  0.497 |
| T2 – T3  Stable medication  Started medication  Stopped medication | *n* = 51  48 (94)  1 (2)  2 (4) | *n* = 47  47 (100)  0 (0)  0 (0) | 2.9  0.9  1.9 | 0.244  1.000  0.496 |
| T3 – T4  Stable medication  Started medication  Stopped medication | *n* = 47  45 (96)  2 (4)  0 (0) | *n* = 43  43 (100)  0 (0)  0 (0) | 1.9  1.9  - | 0.495  0.495  - |
| T4 – T5  Stable medication  Started medication  Stopped medication | *n* = 48  47 (98)  1 (2)  0 (0) | *n* = 45  43 (96)  0 (0)  2 (4) | 0.4  0.9  2.2 | 0.609  1.000  0.231 |
| **Percentage of patients receiving mental health care***** | | | | |
|  | ***n (*%)** | ***n (*%)** | **χ^2^** | ***p*** |
| **T0 – T1** | 45 (82) | 49 (94) | 3.858 | 0.049 |
| **T1 – T2** | 46 (90) | 48 (96) | 1.318 | 0.251 |
| **T2 – T3** | 44 (86) | 46 (94) | 1.605 | 0.205 |
| **T3 – T4** | 42 (84) | 37 (86) | 0.076 | 0.783 |
| **T4 – T5** | 43 (84) | 40 (85) | 0.012 | 0.913 |
| **Number of visits mental health care professional between time points***** | | | | |
|  | ***M* (SD)** | ***M* (SD)** | ***t*** | ***p*** |
| **T0-T1** | 3.4 (5.1) | 4.8 (5.0) | 1.427 | 0.156 |
| **T1-T2** | 3.6 (3.1) | 5.5 (5.3) | 2.147 | 0.035 |
| **T3-T4** | 3.1 (3.7) | 4.2 (4.8) | 1.246 | 0.216 |
| **T4-T5** | 3.5 (4.6) | 4.4 (4.5) | 0.997 | 0.321 |
| *Note:* Only includes patients that completed 1> follow-up measurements  * Includes mood stabilizers, antidepressants, antipsychotics **Medication was considered stable when there were no changes in either specimen or dosage during the entire follow-up period  ***Mental health care includes: psychiatrist, psychologist, psychiatric nurse, social worker | | | | |

**Supplement 3. Mental health care utilization TAU**
