# Supplementary material for: Mindfulness-based cognitive therapy v. treatment as usual in people with bipolar disorder: A multicentre, randomised controlled trial
Source: Psychol Med. 2023 Mar 7;53(14):6678–90. doi: 10.1017/S0033291723000090 (PMC10600813; doi:10.1017/S0033291723000090)
Supplement: Supplementary file 1 [file S0033291723000090sup.zip › S0033291723000090sup004.docx]

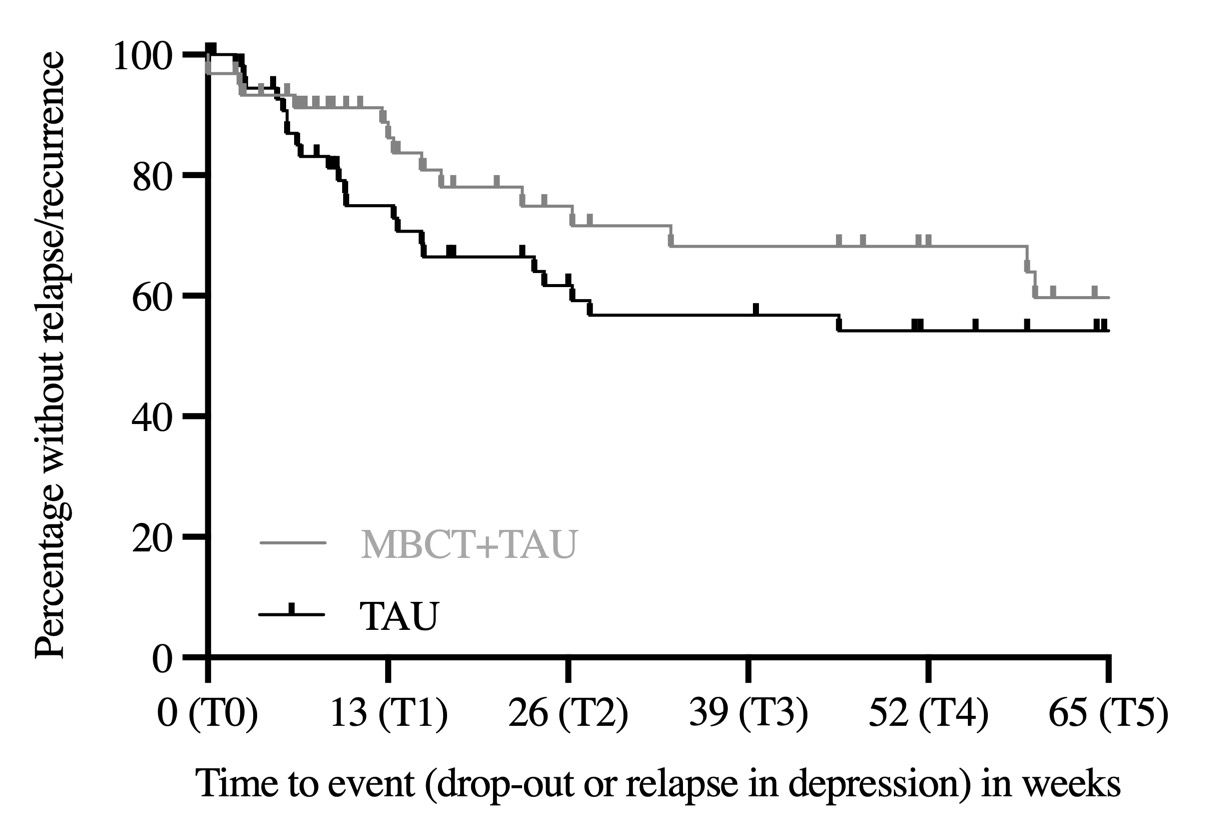


Time to event (drop-out or relapse in depression) in weeks

Percentage without relapse/rcurrence

**Number at risk**

|  | **T0** | **T1** | **T2** | **T3** | **T4** | **T5** |
| --- | --- | --- | --- | --- | --- | --- |
| **MBCT+TAU** | 54 | 33 | 26 | 22 | 18 | 0 |
| **TAU** | 57 | 34 | 26 | 22 | 17 | 0 |

**Supplement 4: Figure 2.** Proportion of patients who did not relapse in depression over 15-months follow-up for MBCT+TAU (*n* = 64) and TAU (*n* = 62)
