# Supplementary material for: Mindfulness-based cognitive therapy v. treatment as usual in people with bipolar disorder: A multicentre, randomised controlled trial
Source: Psychol Med. 2023 Mar 7;53(14):6678–90. doi: 10.1017/S0033291723000090 (PMC10600813; doi:10.1017/S0033291723000090)
Supplement: Supplementary file 1 [file S0033291723000090sup.zip › S0033291723000090sup005.docx]

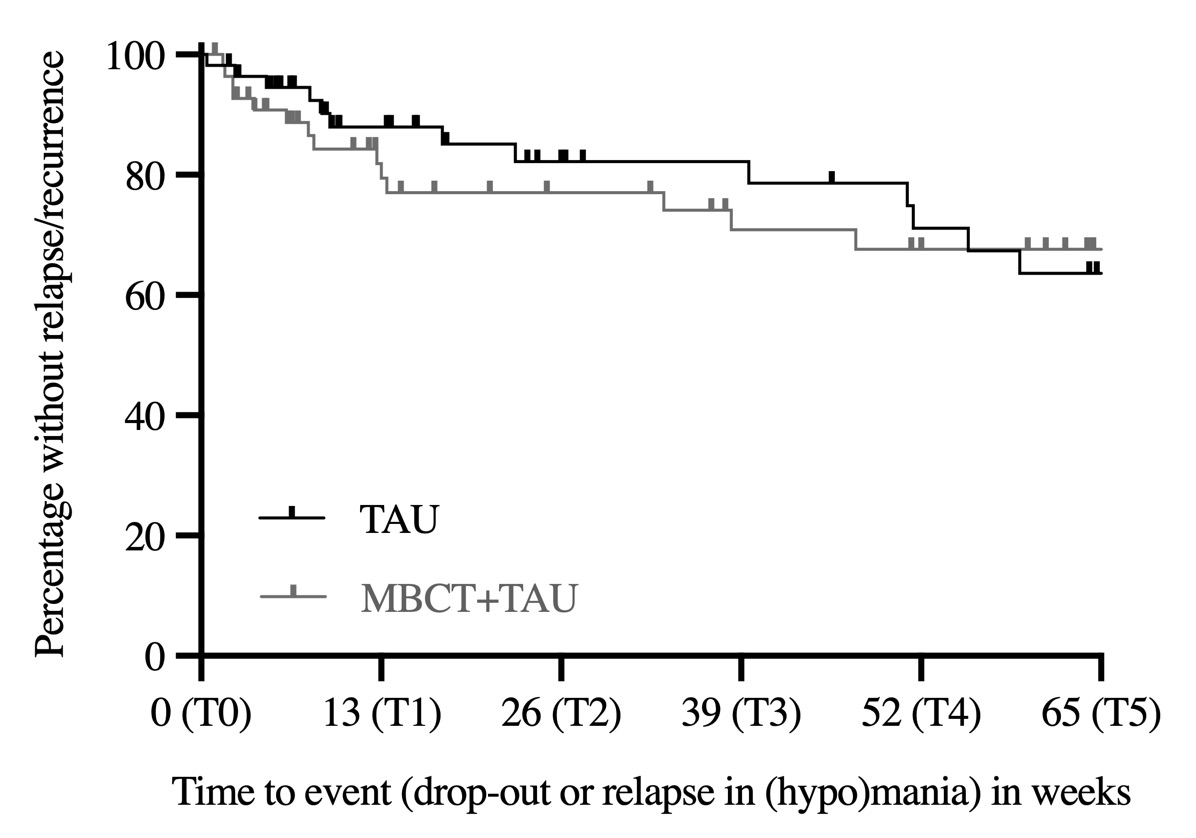


Time to event (drop-out or (hypo)mania) in weeks

Percentage without relapse/recurrence

**Number at risk**

|  | **T0** | **T1** | **T2** | **T3** | **T4** | **T5** |
| --- | --- | --- | --- | --- | --- | --- |
| **MBCT+TAU** | 54 | 32 | 26 | 22 | 17 | 0 |
| **TAU** | 53 | 32 | 25 | 23 | 28 | 0 |

**Supplement 5: Figure 3.** Proportion of patients who did not relapse in (hypo)mania over 15-months follow-up for MBCT+TAU (*n* = 64) and TAU (*n* = 63)
