# Supplementary material for: Mindfulness-based cognitive therapy v. treatment as usual in people with bipolar disorder: A multicentre, randomised controlled trial
Source: Psychol Med. 2023 Mar 7;53(14):6678–90. doi: 10.1017/S0033291723000090 (PMC10600813; doi:10.1017/S0033291723000090)
Supplement: Supplementary file 1 [file S0033291723000090sup.zip › S0033291723000090sup007.docx]

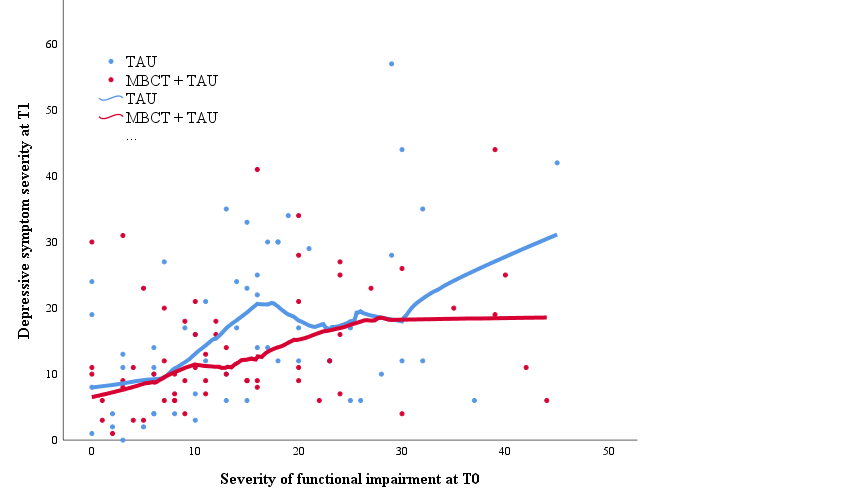


**Supplement 7: Figure 5.** Plot of severity of functional impairment (FAST) at T0 and depressive symptom severity (IDS-C) at T1 with Loess curves for MBCT+TAU and TAU
